# Orthodontic treatment pause during COVID-19 outbreak: Are we overlooking potential harms to our patients and their treatment outcomes?

**DOI:** 10.1590/2177-6709.26.2.e21ins2

**Published:** 2021-04-30

**Authors:** Feras ABED AL JAWAD, Najah ALHASHIMI

**Affiliations:** 1 Orthodontics Section, Hamad Dental Center, Hamad Medical Corporation (Doha, Qatar).; 2 College of Dental Medicine, Qatar University (Doha, Qatar).

**Keywords:** COVID-19, Orthodontic treatment, Harm

## Abstract

**Introduction:**

The global outbreak of coronavirus disease 2019 (COVID-19) has led all dental governing bodies across the world to minimize or cancel routine/elective dental procedures and limit dental services to only acute emergency situations. Orthodontic treatment is a long-term treatment that necessitates several appointments. However, if patients are left unsupervised during this pandemic, serious harms might arise apart from acute orthodontic emergencies.

**Objective:**

In this article we highlight these harms and their negative impact on patients and the overall success of their treatment.

**Conclusion:**

We briefly presented actions needed to be undertaken by orthodontists to gradually allow for recalling patients according to the stage of their treatment to avoid any potential harms to the treatment outcome and avoid any subsequent medicolegal consequences.

## RESUMO

**Introdução:**

O surto global do coronavírus 2019 (COVID-19) levou todos os órgãos diretivos de Odontologia, ao redor do mundo, a minimizar ou cancelar procedimentos odontológicos de rotina/eletivos e limitar os serviços odontológicos apenas a situações de emergência aguda. O tratamento ortodôntico é um tratamento de longa duração que requer várias consultas. No entanto, se os pacientes não forem supervisionados durante essa pandemia, danos graves podem surgir, além das emergências ortodônticas.

**Objetivo:**

No presente trabalho, destacamos esses danos e seu impacto negativo nos pacientes e no sucesso geral de seus tratamentos.

**Conclusão:**

Foram apresentadas, resumidamente, as ações que devem ser realizadas pelos ortodontistas para gradualmente retomar o atendimento dos pacientes, conforme o estágio de seu tratamento, para evitar quaisquer danos potenciais ao desfecho do tratamento e evitar quaisquer consequências médico-legais subsequentes.

## INTRODUCTION

The global outbreak of coronavirus disease 2019 (COVID-19) has dramatically changed the world in all aspects. This has led the World Health Organization (WHO) to declare this disease outbreak as a global pandemic.^1^

The common mode of COVID-19 transmission is either by respiratory droplets or contact routes. As a result, guidelines for healthcare workers started to emerge in an attempt to prevent COVID-19 transmission.^2^ ^,^ ^3^ In the dental profession, it is well known that dental procedures produce aerosols and droplets that pose real risk of disease transmission. Furthermore, coronavirus can remain suspended in the air for longer periods and can contaminate surfaces and instruments that can lead to indirect transmission.^4^

The fact that dental practices are focal points for cross-infection has led all dental governing bodies across the world to minimize or cancel routine/elective dental care, and limit services to only acute emergency situations, relying on telecommunication, pharmaceutical options, the use of high-volume saliva ejectors and avoid the use of aerosol-generating procedures or any procedure that can stimulate coughing. In addition, guidelines have been published during this pandemic to maximize personal protection equipment (PPE) in an effort to reduce the risk of COVID-19 transmission.^2^

Although several vaccines have been recently introduced, it is not known how long COVID-19 will last, given the fact that little is known with respect to its characteristics. Furthermore, there is no effective treatment or medication for this disease, until now, that can aid in reducing severe complications or mortality rate. This in turn, will have negative long-term implications on patients whose care involves several appointments, such as orthodontic patients, who are at the moment experiencing delays in their routine follow-ups.

A recent review summarized advice that can be provided to patients for the short-term management of orthodontic emergencies. Among recommendations presented, were: treatment advice should be delivered first remotely and, only when necessary, in-person treatment can be given, provided strict infection control protocols are implemented.^5^

However, there are other problems that might arise, in addition to acute orthodontic emergencies, which may lead to different types of harm, with respect to treatment outcomes. Such problems are related to the stage of treatment, whether in active or passive phases of orthodontic treatment, and require an urgent need to be highlighted in this unprecedented situation. Therefore, the aim of the present paper is twofold. Firstly, to highlight harms that might arise, other than acute orthodontic emergencies, in unsupervised orthodontic cases, due to global suspension or delay of routine orthodontic follow-ups during the COVID-19 outbreak. Secondly, to briefly present actions needed to be undertaken by orthodontists to gradually allow for recalling patients, according to the stage of their treatment, to avoid any future potential harms to the final treatment outcome. 

## 1. HARMS RELATED TO UNSUPERVISED ORTHODONTIC PATIENTS

Orthodontic treatment is a long-term treatment that necessitates several appointments on a monthly basis or sometimes less, depending on the type of appliance used. To achieve successful treatment outcomes, orthodontists should conduct comprehensive examination, give accurate diagnosis and plan treatment steps efficiently. In addition, every effort should be made to protect the health of both hard and soft oral tissues and the psychological well-being of the patient from any harm.^6^ ^,^ ^7^

The WHO defines patient harm as “an incident that results in harm to a patient, such as impairment of structure or function of the body and/or any deleterious effect arising there from or associated with plans or actions taken during the provision of healthcare, rather than an underlying disease or injury, and may be physical, social or psychological (e.g., disease, injury, suffering, disability and death)”.^8^ Although harms and complications related to orthodontic treatment are not life-threatening and less severe than other medical interventions, it is well reported that they might cause functional, social and psychological impairments to patients.^9^ From an ethical point of view, orthodontists should be aware of all risks expected, and should inform their patients about them including those that are not under the control of the clinician, such as the disruptions of routine orthodontic follow-ups. In addition to adverse effects on patients’ treatment outcomes, disruptions in follow-ups can lead to financial burdens of the whole healthcare system providing any type of medical or dental care.

During the outbreak of COVID-19, several guidelines and recommendations were published with respect to dealing with acute orthodontic emergencies, with no mention of other potential harms that might arise due to unsupervised orthodontic cases. Here, we present examples of potential harms that orthodontists should be aware of to prevent future failure of orthodontic treatment.

### 1.1 HARMS RELATED TO THE UNSUPERVISED MECHANICS FOR TOOTH MOVEMENT

Depending on the stage of treatment, unwanted tooth movement can occur as a result of prolonged period of time without monitoring or adjustments. Unwanted tooth movement can take place in several situations. In extraction cases, if teeth are left without monitoring, space loss is inevitable. Applying orthodontic forces, such as those used for retraction of teeth, torqueing, impacted teeth traction, intrusion, or any other type of tooth movement, if left unsupervised, can lead to serious harms to teeth and associated supporting periodontal tissues. These may include cortical bone perforations, placing teeth in unfavorable position, root resorption and severe tooth rotation and mobility.^10^ This in turn, will jeopardize the treatment goals and will have a negative impact on the final treatment outcome. Therefore, patients who are in an active stage of orthodontic treatment should be evaluated and monitored carefully and be given priority to be seen with high sense of urgency. Clinicians should weight the potential benefits of recalling patients, and the risks of contracting an infection, such as COVID-19, and patients should be informed and involved in the decision making. 

### 1.2 HARMS RELATED TO FINISHED TREATMENT

Retention is one of the most important stages of orthodontic treatment. The aim of this stage is to maintain the stability of the corrected occlusion, as teeth have inherent tendency to return to their original positions.^11^ Retention can be achieved by either fixed or removable retainers. Therefore, careful monitoring, adjustment and assessment of retainers should continue post-treatment, to prevent relapse. It is not uncommon to experience problems related to retainers with respect to patients’ compliance, breakages or losing them.^12^ Usually, the patient is instructed to report any problems related to retainers as soon as they occur. However, during this pandemic, and due to the current regulations, problems related to retainers are not considered an urgent situation that necessitates immediate care. As a result, relapse will take place, with variations in severity depending on the type of malocclusion before treatment. This will lead to serious psychological harm to the patient after complying with long-term treatment and incurring all financial expenses. On the other hand, this will frustrate the orthodontist and will lead to extra treatment time, depending on the extent of the problem. Therefore, problems related to retainers during the COVID-19 outbreak should be regarded as an urgent need for care and be given priority, in order to prevent any sort of relapse. 

### 1.3 PSYCHOLOGICAL HARMS ON PATIENTS

The global outbreak of COVID-19 has imposed changes to our behaviors and the way we interact with the outside world. Restricted access to healthcare facilities is among the important changes that people have to cope with. Unless an emergency situation is present, patients cannot continue with their routine medical or dental follow ups. This issue might lead to distress, panic and immense pressure on patients, in particular, orthodontics patients. The fact that orthodontic follow-ups are discontinued during this outbreak has led many patients to question the progress of their treatment and whether any harm would happen for this long delay. In addition, several studies reported that patients who are undergoing orthodontic treatment will experience positive impact on their quality of life (QoL) during the course of treatment.^13^ Such delay could lead to negative impact on their QoL that might affect their compliance to treatment and their overall expectations about the final outcome. Therefore, it is highly recommended during this pandemic to keep communicating with all patients, even in the absence of an emergency situation, to reduce their anxiety levels and build their confidence level for the coming future. 

### 1.4 HARMS TO TEETH AND PERIODONTIUM

In addition to improving esthetics by correcting jaws relationship and abnormal teeth positions, orthodontists aid in creating a healthy environment to teeth and the supporting tissues. However, in some instances, orthodontic treatment can hold potential harm on the periodontal tissues. This complication could be aggravated if the patient is left unsupervised for long time. Unwanted tooth movements, as mentioned earlier, or not following strict and rigorous oral hygiene measures due to the long wait can lead to periodontal problems and demineralization of teeth - especially in teenagers, who constitute the majority of orthodontic patients.^14^ Therefore, every effort should be made for oral hygiene reinforcement, by contacting patients regularly either by calling them or sending letters or emails. Such practice will eliminate harm to teeth and the periodontium, and will reduce referrals to other dental disciplines, which will lead to fewer burdens on dental practices during this pandemic.

## 2. ACTIONS NEEDED TO BE UNDERTAKEN BY ORTHODONTISTS TO GRADUALLY ALLOW FOR RECALLING PATIENTS, TO PREVENT FAILURE OF TREATMENT AND AVOID ANY MEDICO-LEGAL CONSEQUENCES

What if the patient experienced any type of harm, previously mentioned, and decided to file an official complaint or a lawsuit to the regulatory governing body? Who is to blame? Most Healthcare professionals, including orthodontists, suspended or minimized routine/elective medical interventions, based on international guidelines and recommendations from the WHO and the state/local health regulatory authorities. The dilemma every clinician is facing at the moment is what type of harms, not related to acute emergency situations, are expected, and could they be prevented? This should be balanced against the risk of contracting a pandemic disease such as COVID-19. According to the American Association of Orthodontics (AAO) recent statement:^15^ “The AAO recommends that its members follow all applicable federal, provincial, state and local authorities’ guidance concerning closure recommendations for non-emergency care. To that end, the AAO defines emergency orthodontic care as care that will relieve pain and/or infection, is trauma-related, or is critically necessary to prevent harm to the patient”. This statement implies that, in addition to any type of physical harm the patient might experience, harm from other non-emergency situations should receive great deal of attention and immediate care. 

Jerrold^16^ summarized the actions needed to be taken after few months of orthodontic treatment suspension. He recommends that every orthodontist should do in-depth analysis of every patient record and prioritize patients to be recalled according to the stage of orthodontic treatment. Patients who are under active orthodontic forces that have the potential to move teeth beyond where planned should be recalled, whereas patients in passive stage of treatment or in the retention phase can wait, as harm to them is unexpected. It is down to the judgment of the clinician to assess and evaluate the risks that might arise if the patient is kept unsupervised and what is considered an urgent need for care. 

In summary, and due to the fact that it is not known when this pandemic will end, the following actions should be considered and introduced, in addition to the current recommendations we apply in our orthodontic practices, to prevent any potential harms to both patients and treatment outcomes:

1. Comprehensive revision of all patients’ records should be carried out, to develop a system that will classify them according to their stage of treatment. This will allow identifying patients who need to be seen urgently to prevent future harms, such as unwanted tooth movement or any damage to the retention protocol.
2. In line with the above, patients should be informed and educated about the potential harms they might experience with respect to the whole course of their treatment other than acute emergency situations. Risks related to the progress of their treatment if they are not recalled or risks related to contracting a disease, such as COVID-19, if they agree to be recalled should be clarified and documented. Calling patients or using digital communication tools, such as videos, can aid in triaging patients and get a better insight about their current status. In addition, patients who are in a stage where any type of harm is unexpected should be assured and instructed to keep complying with treatment protocols, including oral hygiene maintenance. This in turn will reduce their anxiety levels during the pandemic and will boost their confidence towards the treatment and the whole healthcare system.
3. State/local health regulatory authorities should take their responsibilities to ensure effective healthcare provision during this pandemic. Among these responsibilities, protecting and defending healthcare professionals from any medico-legal issues that might arise due to suspension of routine and elective dental care, including orthodontic care. Furthermore, every effort should be made to support healthcare professionals in many respects. These include; providing them with the most up-to-date information related to COVID-19 outbreak, ensure the availability of personal protective equipment (PPE), provide regular testing for COVID-19 and give them freedom to judge every case based on the status of the patient, potential risks expected and the totality of circumstances.

## CONCLUSIONS

Orthodontists should be aware of potential harms to treatment outcomes that might occur as a result of unsupervised orthodontic treatment, apart from acute emergency situations. It is not known how long COVID-19 outbreak will last, and therefore orthodontists should consider recalling patients who are in high need for care, depending on their stage of treatment. State/local health regulatory authorities should support and protect clinicians during this unprecedented situation, and patients should be informed and educated about all possible consequences. This in turn will avoid any medico-legal issues and will prevent any jeopardy to the final treatment outcomes.
